# 

**Published:** 1883-01

**Authors:** 


					﻿SÜRGGEMjS'OFFS
VoL-XfXr-
January, 1883.
No. 4i
fK/
SulS
ISTDURY.
®rf.SA M

MiSUÄ.
				

